# Past Experiences for Future Applications of Metabolomics in Critically Ill Patients with Sepsis and Septic Shocks

**DOI:** 10.3390/metabo12010001

**Published:** 2021-12-21

**Authors:** Konlawij Trongtrakul, Chanisa Thonusin, Chaicharn Pothirat, Siriporn C. Chattipakorn, Nipon Chattipakorn

**Affiliations:** 1 Department of Internal Medicine, Faculty of Medicine, Chiang Mai University, Chiang Mai 50200, Thailand; konlawij.tr@cmu.ac.th (K.T.); chaicharn.p@cmu.ac.th (C.P.); 2 Metabolomics Unit, Cardiac Electrophysiology Research and Training Center, Chiang Mai University, Chiang Mai 50200, Thailand; siriporn.c@cmu.ac.th; 3 Center of Excellence in Cardiac Electrophysiology Research, Chiang Mai University, Chiang Mai 50200, Thailand; 4 Cardiac Electrophysiology Unit, Department of Physiology, Faculty of Medicine, Chiang Mai University, Chiang Mai 50200, Thailand; 5 Department of Oral Biology and Diagnostic Sciences, Faculty of Dentistry, Chiang Mai University, Chiang Mai 50200, Thailand

**Keywords:** metabolomics, metabolism, sepsis, septic shock, critically ill patients, diagnosis, prognosis, treatment monitoring

## Abstract

A disruption of several metabolic pathways in critically ill patients with sepsis indicates that metabolomics might be used as a more precise tool for sepsis and septic shock when compared with the conventional biomarkers. This article provides information regarding metabolomics studies in sepsis and septic shock patients. It has been shown that a variety of metabolomic pathways are altered in sepsis and septic shock, including amino acid metabolism, fatty acid oxidation, phospholipid metabolism, glycolysis, and tricarboxylic acid cycle. Based upon this comprehensive review, here, we demonstrate that metabolomics is about to change the world of sepsis biomarkers, not only for its utilization in sepsis diagnosis, but also for prognosticating and monitoring the therapeutic response. Additionally, the future direction regarding the establishment of studies integrating metabolomics with other molecular modalities and studies identifying the relationships between metabolomic profiles and clinical characteristics to address clinical application are discussed in this article. All of the information from this review indicates the important impact of metabolomics as a tool for diagnosis, monitoring therapeutic response, and prognostic assessment of sepsis and septic shock. These findings also encourage further clinical investigations to warrant its use in routine clinical settings.

## 1. Introduction

Sepsis is a clinical syndrome defined as a life-threatening organ dysfunction caused by a dysregulation of the host’s response to infection [1]. Septic shock is a worse condition than sepsis, in which patients need vasopressor administration to maintain their mean arterial pressure over 65 mmHg, in a combination with serum lactate level greater than 2 mmol/L, despite adequate volume resuscitation [1]. Sepsis and septic shock result in systemic abnormalities involved in circulatory, cellular, and metabolic failure [1], and are associated with high mortality rate, which is 25–35% of critical illness [2]. Therefore, early identification and management within a golden period, according to hour-1 bundle [3], are considered crucial to improve patients’ survival. Unfortunately, conventional biomarkers, including procalcitonin and interleukin-6, have a limitation in their sensitivity and accuracy [4]. For this reason, new biomarkers are needed, and metabolomics is currently considered as a new hope for sepsis biomarkers [5].

Metabolomics is one of the omics sciences providing information about low molecular weight chemical compounds inside human biological specimens. This has become increasingly acknowledged in critically ill patients with sepsis and septic shock. However, the complicated alterations of various metabolic pathways in sepsis make it difficult for every single metabolite to serve as the sole biomarker for the clinical practice of sepsis. This article aims to provide an overview of metabolomic-assisted tools for critically ill patients with sepsis and septic shock in order to spot the possibility of applying metabolomics for diagnosis, monitoring of the treatment response, and prognostic evaluation of these two serious conditions. The information from this comprehensive review can contribute to further studies, integrating metabolomics with other molecular modalities and clinical manifestation, leading to the establishment of the use of a metabolomic approach in the clinical settings of sepsis and septic shock, which ultimately results in a reduction in sepsis-related mortality.

The pertinent evidence of metabolomic-assisted tools for critically ill patients with sepsis and septic shock was searched in correspondence with the assigned keywords, including ‘metabolomics, adult critically ill patients, and sepsis OR septic shock’ from PubMed, from its inception to May 2021. The relevant studies from this search were extracted and grouped into five issues, including the application of metabolomics for (1) sepsis diagnosis, (2) septic shock diagnosis, (3) prognostication in sepsis, (4) prognostication in septic shock, and (5) monitoring treatment response in sepsis and septic shock.

## 2. The Role of Microbiota and Its Metabolites in the Development of Sepsis and Septic Shock

Since sepsis and septic shock arise from a systemic inflammatory response to infection [6], microorganisms as infectious agents play a crucial role in the development of sepsis and septic shock. For this reason, microbial-related metabolites can be potential metabolic biomarkers to clarify the development, progression, and prognosis of sepsis and septic shock. These microbial-related metabolites include glycine, alanine, histidine, creatine, phenylalanine, 3-nitotyrosine, glutathione, glucuronic acid, gluconic acid, myoinositol, maltose, ribitol, ribonic acid, 3,4-dihydroxy-butanoic acid, 2,3,4-trihydroxybutyric acid, formic acid, 2-oxoiso-caproate, betaine, acetylacetic acid, stearic acid, oleic acid, linoleic acid, linoleic acid, 4,7,10,13,16-docosapenta-enoic acid, 4,7,10,13,16,19-docosahexaenoic acid, phenylacetic acid, phenylpropionic acid, 4-hydroxy-phenylacetic acid, homovanillic acid, 3-hydroxybutyric acid, 2-hydroxyiso-valeric acid, phenyllactic acid, 4-hydroxy-phenyllactic acid, ethanolamine, taurine, hypotaurine, phosphoethanol-amine, creatinine, proline, indoxyl sulfate, uracil, hypoxanthine, uric acid, pseudouridine, N^1^-methyladenosine, N^2^,N^2^-dimethylguanosine, and N^6^-carbamoyl-threonyladenosine. Basically, all of these metabolites can be categorized into (1) amino acids and their derivatives, (2) polyols and their derivatives, (3) fatty acids and their derivatives, (4) hydroxy acids and their derivatives, (5) amines and nitrogen heterocycles, as well as (6) nitrogen-containing bases of nucleic acids, nucleosides, and their derivatives. The roles of these microbial-related metabolites have been reviewed previously, and more information can be found in that review [7].

## 3. Metabolomics for Sepsis Diagnosis

Despite limited investigation, six studies reported several metabolites which differentiated sepsis patients from their non-septic counterparts (Table 1). The elderly patients were prone to have sepsis than the young patients, and sepsis patients were more severe than those who did not have sepsis, as indicated by a higher APACHE-II score. Serum and plasma are the two common biomaterial samples [8,9,10,11,12,13]; however, one study [13] used erythrocytes for investigation in addition to plasma. Samples were mostly gathered within 24–36 h after the admission to ICU. According to those six reports, the common metabolic pathways that facilitated sepsis diagnosis were (1) amino acids and amines, (2) fatty acid (FA)-related metabolites, and (3) phospholipids.

### 3.1. Alterations of Amino Acids and Amines in Sepsis

It has been shown that most amino acids were decreased during sepsis, as triggered by catabolic hormones, inflammatory mediators, and ubiquitin proteasome system [14]. Using a metabolomic approach, the changes in amino acids as a result of sepsis are comprehensively summarized in Table 1. In sepsis, the levels of cysteine and lysine were decreased [10,11], whereas the levels of glycine, serine, polyamines, and amino acid-derived carnitines were increased due to the body’s response to infection [9,10,12].

#### 3.1.1. A Decrease in Cysteine and Lysine

The level of cysteine and lysine were found to be decreased in sepsis patients [10]. Since cysteine is used for the synthesis of antioxidants glutathione and taurine, a reduction in cysteine suggests an increased requirement of cysteine for antioxidants synthesis to counteract oxidative stress in sepsis [15].

Indeed, lysine is involved in physiological functions for cytokines synthesis, lymphocytes proliferation, nitric oxide (NO) synthesis regulation, anti-viral infection, and anti-inflammation [11,16]. In mice with sepsis, lysine supplementation revealed a lesser degree of inflammation and less hypotensive episodes than those received placebo [17]. However, the knowledge regarding the roles of lysine supplementation in patients with sepsis remains very limited. Future clinical studies are needed to clarify its use in septic patients.

#### 3.1.2. An Increase in Glycine, Serine, Polyamines, and Amino Acid-Derived Acylcarnitines

Glycine has been shown to attenuate inflammatory response [18], and has a cytoprotective property [19]. This metabolite was increased in patients with sepsis [10]. Interestingly, glycine administration was shown to reduce hepatic injury in endotoxic shock [20]. Despite its promising benefits, limited clinical study is available. Therefore, its protective mechanism and safety dose remain to be elucidated [19].

Serine, a precursor for glucose and glycine synthesis, was found to be increased during sepsis [12]. Both glycine and serine are involved in the generation of glutathione, and are necessary for the physiological function of macrophages [21] and T lymphocytes proliferation [22]. An increment of serine in sepsis patients could be a compensatory process to counteract sepsis-induced oxidative stress [16,21].

The level of polyamines, including spermidine and spermine, were increased in patients with sepsis [12]. Two studies revealed serum [23] and polyamines, originally synthesizing from the arginine–ornithine pathway [24], to be involved in the RNA synthesis, transcription, and translation of human cells [25,26], as well as in several pathogens [24,27]. However, the interaction between host cells and the pathogen’s polyamine requires elucidation. Moreover, polyamines might be markers for sepsis severity. Therefore, the correlation between polyamines levels and sepsis severity should be further investigated.

Patients with sepsis were also found to have increased levels of short-chain acylcarnitines (ACs), including C3 to C5 carnitines [9]. These ACs are derived mainly from branched-chain amino acids (BCAAs), which suggest an enhancement of BCAA oxidation [28] and are markers of insulin resistance [29]. Interestingly, the interplay between sepsis and insulin resistance is most commonly encountered in order to provide sufficient glucose for a cellular consumption [30]. Thus, short-chain ACs may represent the state of insulin resistance induced by sepsis as well.

### 3.2. Alterations of Fatty Acids and Their Related Metabolites in Sepsis

In sepsis, eicosanoid FAs were decreased [13]. In contrast, free monounsaturated fatty acids (MUFAs) that degrade from the cell membrane phospholipids were increased [13]. Moreover, free FA-derived ACs and ceramides were altered [8,9,12].

#### 3.2.1. A Decrease of Eicosanoids

The circulating phospholipase A2 (PLA2) mediates a release of proinflammatory lipids called eicosanoids from the cell membrane phospholipids [31]. Eicosanoids are composed of arachidonic acid (AA) and polyunsaturated fatty acids (PUFAs) that have multiple regulatory functions involved in the inflammatory process [31]. A significant decrease in some n-3 PUFAs, including docosapentaenoic acid (C22:5 n-3) and docosahexaenoic acid (C22:6 n-3), was revealed [13]. These are the precursors of anti-inflammatory mediators, called resolvins. Hence, a greater reduction in eicosanoids may reflect the degree of sepsis severity, on which a future study should be conducted to identify this relationship.

#### 3.2.2. An Increase in Free Fatty Acids

A noticeable increase in MUFAs in patients with sepsis was exhibited, particularly oleic acid (18:1 n-9) [13]. This increment may be as a result of sepsis-induced lipolysis [32].

#### 3.2.3. An Alteration of Fatty Acid-Derived Acylcarnitines and Ceramides

The major function of carnitines is to facilitate the transportation of long-chain fatty acids (LCFAs) from cytoplasm into the mitochondria for β-oxidation [28]. Moreover, carnitines can remove an overwhelming amount of incomplete oxidized FAs in order to prevent the intoxication of acyl-CoA [28]. In other words, increased medium-chain ACs represent increased incomplete β-oxidation. It was observed that there were alterations of ACs in patients with sepsis compared to those without sepsis. These changes include a decrease in long-chain ACs–C16:2(OH) carnitine [12], and an increasing level of medium-chain ACs–C6 [9,12], C8 [9], and C10 carnitines [9]. These findings suggested that sepsis is associated with decreased FA uptake into the mitochondria and increased incomplete β-oxidation.

Ceramides, the initial product of sphingomyelins (SMs), are another bioactive FA that play a role in the regulation of immune cells, autophagy, and apoptosis [33]. The structure of ceramide looks similar to bacterial lipopolysaccharides, which involves the pathogenesis of sepsis [34]. A reduction in C23:0 and C24:0 and an increment in C16:0, C18:0, C20:0, C22:1, and C24:1 ceramides were found in sepsis patients [8]. Additionally, an increase in the ratio of total ceramide-to-SM, as well as some specific ceramides to their SM precursor molecules, including C16:0, C18:0, C20:0, C22:0, C22:1, C23:0, C24:0, and C24:1, were revealed [8]. Indeed, increased ceramide levels could be considered to be an indicator for sepsis diagnosis.

### 3.3. Alterations of Phospholipids in Sepsis

Phospholipids are well known for a major component of cell membrane [35]. Several kinds of phospholipids are decreased in sepsis, including sphingomyelins (SMs) [13] and lysophosphatidylcholines (lysoPCs) [8,12,13], whereas cardiolipins [13] are increased. However, phosphatidylcholines (PCs) are contradictorily changed [12,13].

#### 3.3.1. A Decrease in Sphingomyelines and Lysophosphatidylcholines

The level of SMs were found decreased in both plasma and erythrocyte of patients with sepsis, which included C34 to C44 SMs [13]. It is well known that inflammation triggers SM catabolism via the activation of acid sphingomyelinase enzymes, leading to increased downstream metabolites of SM, such as ceramides and PCs [36].

LysoPCs play a role in inflammation via the regulation of several immune cells, including macrophages and monocytes [37]. C16:0, C18:0, C18:1, and C18:2 lysoPCs, as well as their molar ratios corresponding to their precursor PCs, were decreased [8]. Consistently, another two studies demonstrated a reduction in lysoPCaC14:0 [12], C24:0 lysoPCs [12], and C15 to C20 lysoPCs [13]. These results suggest that it might be related to the presence of lysophospholipase D and autotaxin enzymes that hydrolyze lysoPCs [38].

#### 3.3.2. An Increase in Cardiolipins

Cardiolipins are an essential lipid required for mitochondrial respiration, which are found almost in the inner membrane of mitochondria [39]. Cardiolipin was reportedly increased, as indicated by a ratio of 1′[18:0/18:2]-to-3′[20:0/10:0] cardiolipin [13]. This increment reflects a cardiolipin translocation outside of a damaged mitochondria in sepsis [40].

#### 3.3.3. An Alteration of Phosphatidylcholines

The levels of some PCs, including PCaa C32:2 [12], PCae C36:6 [12], PCae C40:4 [12], PCae C42:6 [12], PCae C44:4 [12], C16:0/20:1 PC [13], and C16:0/20:3 PC [13] were found decreased in patients with sepsis. However, the contradicted results were demonstrated. Indeed, PCaa C32:0 [9], PCae C34:1 [9], PCae C34:2 [9], PCae C36:1 [9], PCae C34:1 [9], C15:0/18:2 [13], C16:0/18:1 [13], C16:0/18:2 [13], C16:0/20:4 [13], and C16:0/20:5 [13] PCs were increased in patients with sepsis. PCs are one of the most abundant phospholipids in all cell membranes [41]. Hence, the alteration of PCs may represent the severity of sepsis-induced cellular dysfunction. In fact, it is possible that PCs can be used as a decision-making guide for early sepsis management in order to decrease the risk of sepsis-induced organ injury.

## 4. Metabolomics for Septic Shock Diagnosis

Two studies revealed serum and plasma [23,42] metabolites that could discriminate septic shock patients from sepsis patients without shock, as listed in Table 2. These metabolites were amino acids and amines, as well as glycolysis-related metabolites.

### 4.1. Alterations of Amino Acids and Amines in Septic Shock

A reduction in BCAAs [23,42] and urea cycle-related amino acids, including glutamine [23], glutamate [23,42], and arginine [23,42], were revealed in septic shock patients. On the other hand, aromatic amino acids (AAAs) [23,42] and proline [23,42] were increased.

#### 4.1.1. A Decrease in Branched-Chain Amino Acids, Glutamine, Glutamate, Arginine, and Proline

BCAAs, including leucine, isoleucine, and valine, are categorized as essential amino acids that have a protein anabolic effect, and elicit wound healing promotion, hepatic encephalopathy prevention, and insulin secretion stimulation [43]. Two studies reported a reduction in BCAAs in their septic shock patients [23,42]. However, current evidence does not support BCAAs’ supplementation in sepsis patients owing to controversial outcomes [43,44]. Therefore, BCAA supplementation in critically ill patients may be resolved by metabolomics study, since metabolomics may help identify the metabolism mediating the effect of BCAA supplementation on sepsis.

Glutamine is an essential amino acid that plays a role in immune function, glutathione production, and the biosynthesis of purines and pyrimidines [45]. A decrement in glutamine in septic shock patients was demonstrated from a single study [23]. This finding may reflect increased glutamine consumption that exceeds the rate of biosynthesis in sepsis [43,45].

Glutamate has functions for preserving nitrogen balance in skeletal muscle [45] and promoting the clearance of nitrogen waste products in the liver before excretion via the kidney [46,47]. Moreover, glutamate can convert into α-ketoglutarate, which is one of the energy sources in the TCA cycle. Both studies revealed a decrease in the glutamate level of their septic shock patients [23,42], which is likely to be a result of low dietary intakes and hepatic glutamate synthesis, as well as an increase in glutamate consumption [46,48].

Arginine, one of intermediate amino acids in the urea cycle, has a pertinent role in the biosynthesis of protein, NO, creatine, urea, and polyamines [49,50]. A decrease in the arginine levels of septic shock patients was demonstrated in both studies [23,42]. Although a low level of arginine is associated with sepsis, its supplementation remains a controversial issue [51]. Indeed, some benefits were shown in trauma and critically ill surgical patients [52]. However, in sepsis shock patients, worsening hemodynamic instability is aggravated, as arginine can turn into NO [49,50].

Proline functions to regulate the cellular redox state, promote lymphocytes proliferation, eradicate pathogens via superoxide formation, advocate wound healing, and is involved in ornithine and polyamine formation via pyrroline-5-carboxylate (P5C) [16]. Proline was decreased in septic shock patients from both studies [23,42]. This result might represent the promotion of tissue repair during sepsis [53].

#### 4.1.2. An Increase in Aromatic Amino Acids

Phenylalanine, tyrosine, and tryptophan are categorized as AAAs [54,55]. Phenylalanine has an important function for producing tetrahydrobiopterin (BH4) cofactor, which is involved in arginine–NO synthesis [56,57]. Although the increment in phenylalanine was reported in septic shock [23,42], the information of tyrosine and tryptophan alteration remained unknown. For this reason, a metabolomics study quantifying a whole AAA metabolic pathway in sepsis and septic shock should be established.

### 4.2. Alterations of Glycolysis-Related Metabolites in Septic Shock

Hypoglycemia was found in both studies [23,42]. This phenomenon could be a result of low dietary intake, a decrease in gluconeogenesis, the depletion of glycogen, or an increase in peripheral consumption [32]. Furthermore, a decrease in mannose and an increase in sucrose and myo-inositol were found [23,42]. Although it is difficult to explain the exact role of these sugars, the alterations may be modulated by the organism, rather than the body’s response to infection.

Acetylcarnitine (C2 carnitine) represents a cycling of acetyl-CoA, which is a product of glycolysis [58]. C2 carnitine was found elevated in patients with septic shock [23,42], owing to an excess acetyl group from metabolic stress that may intoxicate the cell [59].

## 5. Metabolomics for Prognostication Patients with Sepsis

Nine studies reporting numerous metabolites that could prognosticate patients with sepsis non-survivors at 48 h after the ICU admission through day 90 [8,10,11,60,61,62,63,64,65,66]. Almost patients had sepsis, non-survivors were older [8,10,11,61,62,64,65,66], with a greater severity of illness [10,11,60,61,62,63,64,65,66]. Plasma was a specimen of choice for a targeted metabolomic approach [8,11,60,61,62,63,64,66]. The metabolites that help prognosticate non-survivors with sepsis are listed as the following.

### 5.1. Alterations of Amino Acids and Amines in Sepsis Non-Survivors

It was shown that taurine, tryptophan, glutamate, arginine, and serine were decreased in non-surviving patients with sepsis [11]. In contrast, intermediate metabolites of amino acids, including *S*-(3-methyl-butanoyl)-dihydrolipoamide-E [10], amino acid-derived carnitines [61,62], symmetric dimethylarginine (SDMA) [61,66], and asymmetric dimethylarginine (ADMA) [66], were found to be increased in non-surviving patients with sepsis. The details are shown in Table 3.

#### 5.1.1. A Decrease in Taurine, Tryptophan, Glutamate, Arginine, and Serine

Taurine, a sulfur-containing amino acid that most abundant in leukocytes, is recognized as an antioxidant, and exerts an antimicrobial effect [16,67]. Moreover, a decreased level of taurine was associated with hyperlactatemia and cardiopulmonary dysfunction [68]. One study reported a reduction in taurine in non-surviving patients with sepsis at day 28 [11]. Nonetheless, taurine supplementation in sepsis patients revealed promising results in terms of decreasing IL-6 and improving clinical outcomes [69].

One study showed a decreased level of tryptophan associated with sepsis mortality at day 28 [11]. A reduction in tryptophan reflects the increased tryptophan degradation to be kynurenine owing to cytokines release [70], and is considered to be one of the main mechanisms of hypotension in sepsis [71]. Correspondingly, a study showed an increased level of kynurenine in patients with sepsis non-survivors at day 28 [62]. Taken together, a decrease in tryptophan and an increase in kynurenine level may be potential factors with which to determine the severity of sepsis. Moreover, tryptophan can be metabolized to be serotonin and melatonin [72], which attenuate the inflammatory response and play a significant role in the regulation of mood, circadian rhythm, and sleep [73]. A supplementation of tryptophan in critical illness may decrease the incidence of sleep deprivation and delirium, which are common problems in critically ill patients [74]. Thus, future studies are needed to answer this question.

Glutamate, arginine, and serine were decreased in sepsis non-survivors at day 28 [11]. Interestingly, serine alteration was different between study populations. It was decreased in non-surviving patients with sepsis at day 28 [11] but was increased in patients with sepsis, as previously mentioned in Section 3.1.2 [12]. An overwhelming utilization of serine in sepsis non-survivors might explain this finding.

#### 5.1.2. An Increase in *S*-(3-Methyl-butanoyl)-dihydrolipoamide-E, Amino Acid-Derived Acylcarnitines, and Symmetric Dimethylarginine and Asymmetric Dimethylarginine

*S*-(3-methyl-butanoyl)-dihydrolipoamide-E is an intermediate metabolite of leucine degradation [75]. One study demonstrated an increase in this metabolite in non-surviving patients with sepsis within 48 h of admission [10]. Moreover, an increase in short-chain ACs: C3, C4, C5, C5-OH, and C5:1 carnitines were observed in non-surviving patients with sepsis at day 28 [61,62]. These increments may be related to an abnormality in BCAA synthesis and degradation. However, further metabolomic studies covering all metabolites in BCAA synthesis and short-chain ACs production are needed to identify which steps of BCAAs are disrupted in septic non-survivors.

SDMA and ADMA are residual from a broken-down arginine process [76] and are considered as competitive inhibitors of NO synthesis [77]. Based on mortality at day 28, prior studies demonstrated a higher level of SDMA and ADMA at day 1 [61,66], 3 [66], and 7 [66] in sepsis non-survivors than the survivors. These results might suggest an excessive production of NO in septic shock non-survivors.

### 5.2. Alterations of Fatty Acids and Their Related Metabolites in Sepsis Non-Survivors

Non-surviving patients with sepsis at day 7 had an increase in several lipid mediators [60]. Specifically, AA-derived FAs, including prostaglandin F2α and leukotriene B4, and specialized pro-resolution lipid mediators including 17R-protectin D1, resolvin D5, and resolvin E1, were persistently elevated along a study period of 7 days [60]. It is well known that lipid mediators, particularly prostaglandin and leukotriene, enhance vasodilatation and inflammation [78], which may ameliorate by non-steroidal, anti-inflammatory drugs [79,80]. However, a vulnerability to acute kidney injury in critically illness [81,82] limits the feasibility of using NSAIDs in the case of most septic patients. Further study is needed to establish the appropriate medications to counteract the devastated lipid mediators.

In addition, non-surviving patients with sepsis at day 28 had an increase in FA-derived ACs, including medium-chain ACs (C6, C8, C8:1, and C12 carnitines) and long-chain ACs (C16 and C18 carnitines) [61]. The results suggested that sepsis non-survivors have an increased FA uptake, but incomplete β-oxidation [83].

### 5.3. Alteration of Phospholipids in Sepsis Non-Survivors

Several phospholipids were decreased in patients with sepsis non-survivors at day 28 including C22:0 and C24:0 lysoPCs [64], and 1-arachidonoyl-glycerophosphoethanolamine (20:4) [62]. However, phosphatidylglycerol (22:2(13Z,16Z)/0:0) and glycerophosphocholine (GPC) were found to be increased in the non-survivors at 48 h after the ICU admission [10].

### 5.4. Alterations of Glycolysis-Related Metabolites in Sepsis Non-Survivors

Pyruvate and lactate were increased in sepsis non-survivors at day 28 [64]. Instead of entering into the TCA cycle, pyruvate accumulation can convert to lactate when tissue is confronted with poor perfusion [84].

### 5.5. Alterations of Aromatic Microbial Metabolites in Sepsis Non-Survivors

It was demonstrated that plasma 3-(4-hydroxyphenyl) lactic acid was positively associated with death from sepsis at day 28 [62]. This metabolite is classified as an aromatic microbial metabolite that has been shown to be involved in the pathogenesis of septic shock [85]. Therefore, these reports emphasize the impact of microbiota on the development and progression of sepsis.

## 6. Metabolomics for Prognostication Patients with Septic Shock

Meaningful metabolites that can prognosticate septic shock non-survivors since ICU admission to 1 year after hospital discharge are reported from seven studies (Table 4) [42,86,87,88,89,90,91]. Most of the studies demonstrated that non-survivors were older and severer than those survivors [86,87,88,90,91]. Serum or plasma remained the samples for a targeted metabolomics study [42,86,87,88,89]. Furthermore, some studies revealed a dynamic change in metabolite levels over the study period [88,89]. The metabolites that helped prognosticating septic shock non-survivors including amino acids and amines, fatty acids, phospholipids, glycolysis-related metabolites, and TCA cycle metabolites.

### 6.1. Alterations of Amino Acids and Amines in Septic Shock Non-Survivors

Dimethylamine [42] and citrulline [87] and were observed decreasing in septic shock non-survivors. In contrast, SDMA [89], total DMA [89], and tyrosine [89], were increased in septic shock patients with non-survival. Interestingly, the discrepancy regarding phenylalanine [86,87,91] and methionine [86,87,90,91] were revealed among studies.

#### 6.1.1. A Decrease in Dimethylamine and Citrulline

One study revealed a decreased level of dimethylamine in septic shock non-survivors [42]. Dimethylamine is originated from fish and seafood consumption and it is a byproduct of the post-translation modification of arginine that involved in the inhibition of NO synthesis [76,92]. Therefore, future research might be conducted to identify the benefit of dimethylamine supplementation in septic shock patients.

Another study reported that a decrement of citrulline at baseline could predict septic shock non-survivors at day 7 [87]. This low level of citrulline is likely due to a decrease in citrulline absorption through the gastrointestinal tract [49]. Or it might be related to a low level of ornithine and arginosuccinate [87], which involved in citrulline metabolism in the urea cycle pathway. These results suggested that the disturbance of urea cycle can be a potential indicator of septic shock with non-survival.

#### 6.1.2. An Increase in Symmetric Dimethylarginine, Total Dimethylarginine, and Tyrosine

A prior study revealed that SDMA and total DMA, competitive inhibitors of NO synthesis, were increased in septic shock non-survivors at day 28 [89]. Hence, measuring these metabolites could be used prognosticating the patients with septic shock outcomes.

Tyrosine is a precursor for catecholamine biosynthesis under the activity of specific enzyme-phenylalanine hydroxylase (PAH) [16,55]. Two studies demonstrating an increase in tyrosine level as a predictor of the septic shock non-survivors at 24 h after ICU admission [86] and at day 28, respectively [89]. This phenomena might be explained by the fact that PAH function is impaired during sepsis [55].

#### 6.1.3. An Alternation of Phenylalanine and Methionine

The alternation of phenylalanine in septic shock non-survivors were controversial. Indeed, previous studies revealed a decreased phenylalanine in septic shock non-survivors at day 30 [90] and at one year [91]. On the contrary, other studies showed an increased phenylalanine in the non-survivors at 24 h [86] and at day 7 [87]. Since phenylalanine and tyrosine are involved in the same pathway, a whole pathway study may be further investigated in order to justify how phenylalanine interacts in septic shock non-survivors.

Methionine, one of the sulfur-containing amino acids, is a precursor for the syntheses of homocysteine, cysteine, glutathione, creatine, and polyamines [93]. A prior study displayed a decrease in methionine in non-surviving patients with septic shock at day 30 [90]. Furthermore, a decrease in methionine day 7/day 1 ratio in sepsis non-survivors at day 28 were observed [89]. However, one study demonstrated an increased level of methionine in septic shock non-survivors at 24 h [86]. Therefore, a future study quantifying the levels of methionine and its related metabolites should be conducted in order to clarify the inconsistent findings of methionine among studies.

### 6.2. Alterations of Fatty Acid-Related Metabolites in Septic Shock Non-Survivors

In non-surviving patients with septic shock at day 7, the level of long-chain ACs, including C16 and C18 carnitines, were decreased [87]. On the contrary, the level of medium-chain ACs, including C6, C10, and C12 carnitines, were increased [87]. These results indicating a decrease in FA uptake into the mitochondria and incomplete β-oxidation of long-chain FAs, which can be one of the potential markers of septic shock mortality.

### 6.3. Alterations of Phospholipids in Septic Shock Non-Survivors

Several phospholipids were involved in patients with septic shock non-survivors. A reduction in C18:0, C18:2, C20:4, and C20:5 lysophosphatidylethanolamines (lysoPEs) were found in septic shock non-survivors at day 7 [87]. LysoPE is a minor component of the cell membrane lysophospholipids where physiological significance remains undetermined [94]. In addition, a decrease in lysoPCa C16:0, C18:0, and C24:0 was also displayed in septic shock non-survivors at day 28 [88]. However, an increase in C14:0 and C24:0 lysoPCs was reported in other studies [87,88].

Two studies discovered an alternation of several PCs in septic shock non-survivors [88,89]. The non-survivors at day 28 had a decrease in PCaa C32:3, PCaa C34:4, PCaa C36:4, PCae C34:3, and PCae C42:4 [88], and a ration from day 7 by day 1 of PCaa C40:6, PCaa C42:6, PCaa C42:2, PCae C32:2, and PCae C42:5 [89]. Furthermore, some of these PCs had been decreased in the non-survivors at day 90 as well [89]. However, the latest study mentioned an increment in PCae C30:1 in the non-survivors [89]. These findings suggest that several PCs can be used as prognosticating biomarkers for septic shock patients.

### 6.4. Alterations of Glycolysis-Related Metabolites and TCA Cycle Metabolites in Septic Shock Non-Survivors

Hyperglycemia is commonly present in sepsis to provide sufficient glucose for neurons and leucocytes [30]. Glucose was found to be increased in patients with septic shock non-survivors at day 30 [90]. However, hypoglycemia can also be detected in sepsis and is associated with a lethal outcome as well [32].

Lactate was found to be increased in septic shock non-survivors within 24 h [86] and at day 7 [87]. Moreover, pyruvate was also increased in the non-survivors at day 7 [87]. Acetylcarnitine (C2 carnitine) was decreased in patients with septic shock non-survivors at day 7 [87]. This finding is in contrast to that mentioned in Section 4.2, where septic shock patients had an increased acetylcarnitine compared to the non-septic shock patients [23,42]. These contradictory results may be explained by a failure of the metabolic flexibility of acetylcarnitine to switch as fuel sources during sepsis [28].

As a result of mitochondrial dysfunction in sepsis [95], multiple TCA cycle metabolites including citrate, α-ketoglutarate, succinate, fumarate, and malate were increased in septic shock non-survivors within 24 h and at day 7 [86,87]. These metabolites represent an augmentation of aerobic metabolism during sepsis, which promotes oxidative phosphorylation and releases of reactive oxygen species (ROS) [96,97]. The ROS may be one of several reasons for sepsis-induced mitochondrial dysfunction [98].

## 7. Metabolomics for Monitoring Treatment Response in Sepsis and Septic Shock

Two studies have revealed metabolomics that monitor the treatment response in sepsis population [99,100]. The first study aimed to identify patients who would reduce the use of vasopressor after l-carnitine supplementation using metabolomics study [99]. A good response was found in patients with a low ketotic state (3-hydroxybutyrate < 153 μM), where methionine, lysine, phenylalanine, and tyrosine were found to be increased after l-carnitine was given [99]. In addition, patients with a good response had a low level of carnitine and acetylcarnitine as well [99]. The second study characterized septic shock patients who had a sequential organ failure assessment (SOFA) score of lower than 8 or a drop of more than 5 within 48 h after treatment [100]. An untargeted metabolomics comparing the degree of metabolites changes between the responders and non-responders overtime (from baseline to 48 h) found that myristic acid and oleic acid were more decreased, whereas creatinine was less decreased in the responders than that of the non-responders [100]. From targeted metabolomics, alterations of several metabolites showed differently. When comparing the degree of metabolites changes overtime, kynurenine was increased in the responders but lower than of the non-responders. Interestingly, several phospholipids had a predictive ability to determine treatment response. Indeed, most SMs, SM(OH)s, and PCs were increased in the responders, whereas they were decreased in the non-responders. In addition, SM C16:1 and C16-C20 lysoPCs were both increased in both groups; however, a greater increment was found in the responders than in the non-responders [100]. These findings suggest that several metabolites can be potential markers for monitoring the treatment response in sepsis patients.

## 8. Conclusions

Metabolomics is about to change the world of sepsis biomarkers. Not only can it be utilized for sepsis diagnosis, but also for prognosticating and monitoring the therapeutic response. In this review, we found that sepsis can lead to the alteration of enormous metabolites.

**Figure 1:**
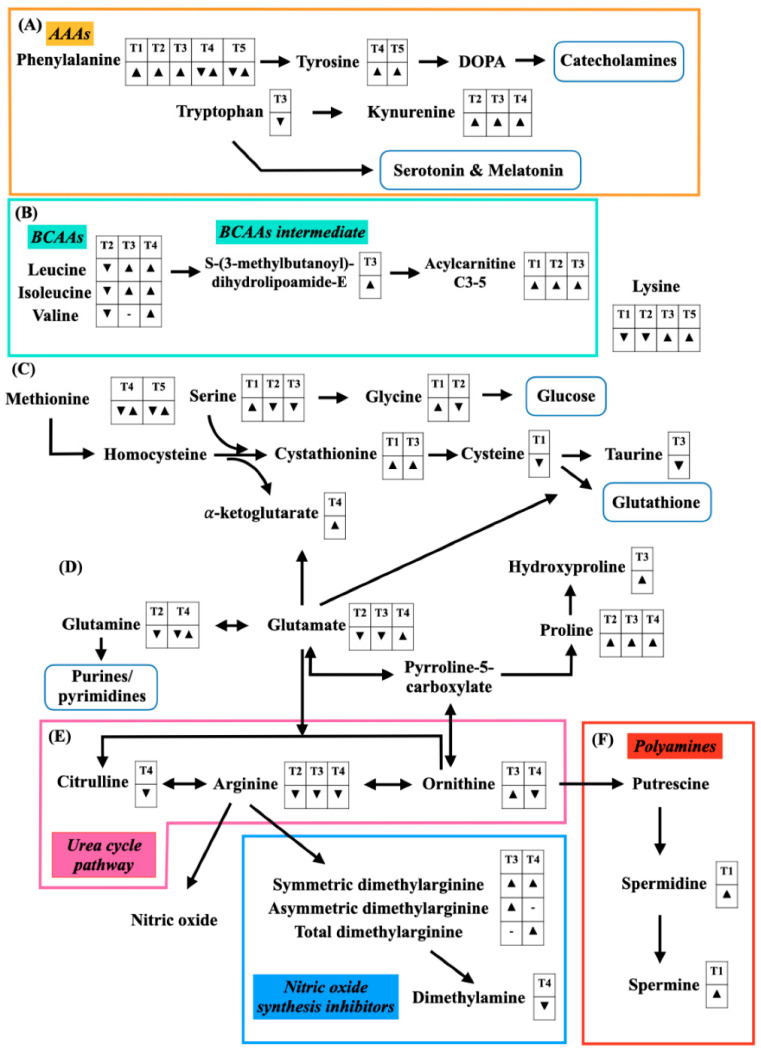
Alterations of amino acids and amines for sepsis diagnosis (Table 1), septic shock diagnosis (Table 2), prognostication of sepsis (Table 3), prognostication of septic shock (Table 4), and monitoring the treatment response (Table 5). A down-sided triangle (▼) represents a decreased level, whereas an up-sided triangle (▲) represents vice versa. (**A**) Aromatic amino acids (AAAs) and its down-stream amino acid are illustrated. Phenylalanine is converted to tyrosine before metabolizing to dihydroxyphenylalanine (DOPA) and catecholamines, respectively. Tryptophan is another AAA that can change to either kynurenine or serotonin and melatonin. (**B**) Branched-chain amino acids (BCAAs) including leucine, isoleucine, and valine are catabolized to *S*-(3-methylbutanoyl)-dihydrolipoamide-E and amino acid-derived acylcarnitines (C3-5), respectively. (**C**) A substrate of glutathione synthesis begins with methionine that converts into homocysteine, cystathionine, and cysteine, respectively. Moreover, serine is involved in the cystathionine production with an exchange of α-ketoglutarate. Cysteine can also turn into taurine, which has an anti-oxidant effect. (**D**) Glutamate is an intermediate substrate between glutathione production and urea cycle-related metabolites. Glutamate can be converted into glutamine and pyrroline-5-carboxylate (P5C). The latter metabolite is a precursor for proline synthesis. Moreover, glutamate can interchange with the urea cycle pathway metabolites. (**E**) The urea cycle pathway metabolites included citrulline, arginine, and ornithine. Arginine is a key amino acid for nitric oxide (NO) synthesis and NO inhibitors, including symmetric dimethylarginine (SMDA), asymmetric dimethylarginine (ADMA), total dimethylarginine (total DMA), and dimethylamine. (**F**) Polyamines are converted from ornithine. The polyamine metabolites include putrescine, spermidine, and spermine.

Some significant amino acids, according to the study populations, are summarized in Figure 1. Multiple amino acids can be used as operational markers for sepsis determination, for instance, arginine, proline, and kynurenine, whereas BCAAs can be used as predictive markers to identify patient survival.

**Figure 2:**
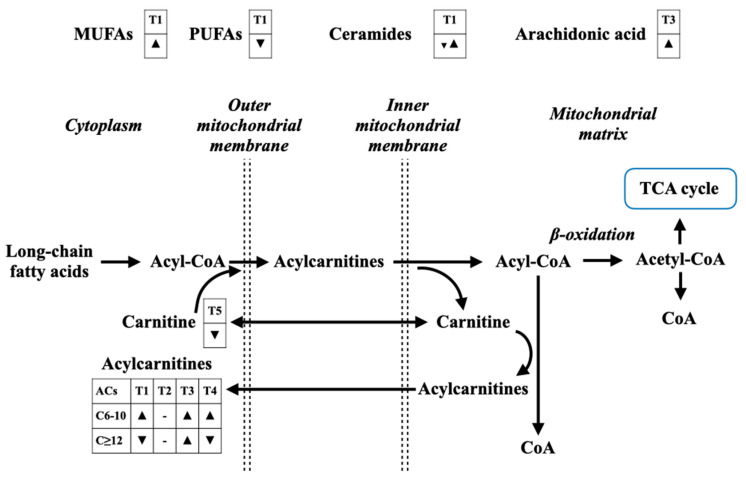
The alterations of fatty acids and fatty acid-related metabolites for sepsis diagnosis (Table 1), septic shock diagnosis (Table 2), prognostication of sepsis (Table 3), prognostication of septic shock (Table 4), and monitoring the treatment response (Table 5). A down-sided triangle (▼) represents a decreased level, whereas an up-sided triangle (▲) represents vice versa. Monounsaturated fatty acids (MUFAs) are found increased in sepsis diagnosis, whereas polyunsaturated fatty acids (PUFAs) are found contractedly. Most of ceramides are increased, together with an increase in arachidonic acids. Long-chain fatty acids enter mitochondrial for fatty acid oxidation (β-oxidation) under the carnitine shuttle process. The final product of β-oxidation is acetyl-CoA that can enter TCA cycle for an energy production. However, mitochondrial dysfunction in sepsis can alter β-oxidation process, leading to an accumulation of medium-chain acylcarnitines in the cytoplasm and in the circulation, which indicates incomplete β-oxidation.

Free FAs are summarized in Figure 2. These metabolites also present as sepsis biomarkers as well; however, a further study combining FAs with other connecting metabolic pathways, in particular FA-derived ACs and TCA cycle metabolites, with a step-by-step metabolomic approach in each of these pathways, is needed.

**Figure 3:**
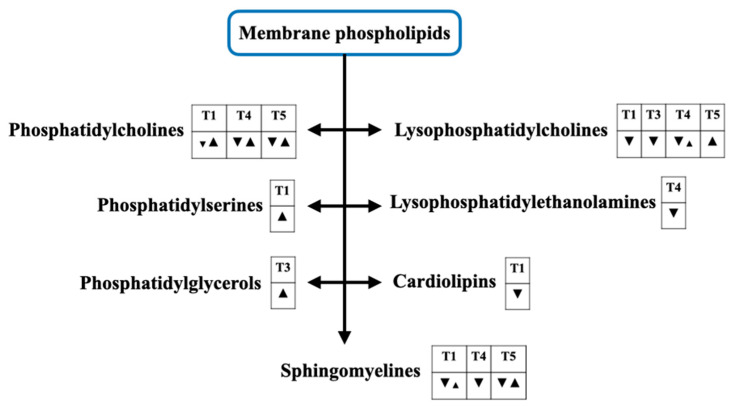
(Previous page) The alterations of cell membrane phospholipids for sepsis diagnosis (Table 1), septic shock diagnosis (Table 2), prognostication of sepsis (Table 3), prognostication of septic shock (Table 4), and monitoring the treatment response (Table 5). A down-sided triangle (▼) represents a decreased level, whereas an up-sided triangle (▲) represents vice versa. Several kinds of the cell membrane phospholipids are involved in this setting. An alteration of phosphatidylcholines (PCs), phosphatidylserine (PS), phosphatidylglycerols (PGs), lysophosphatidylcholines (LysoPCs), lysophosphatidylethanolamines (LysoPEs), cardiolipins, and sphingomyelines (SMs) are demonstrated.

Figure 3 demonstrates phospholipid-related membrane phospholipids, as sepsis always interferes in this structure. For this reason, any interventions that can specifically protect the cell membrane injury by septic pathogens may be established under the guide of the levels of these metabolites.

**Figure 4:**
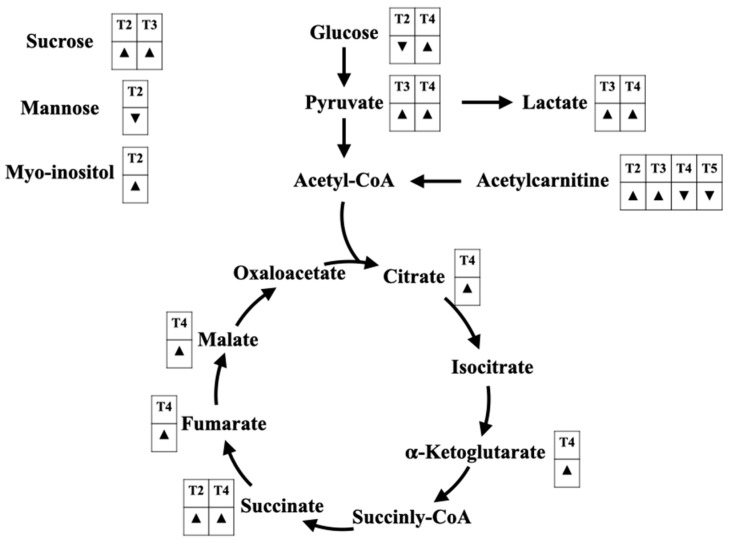
The alterations of glycolysis-related metabolites and tricarboxylic acid (TCA) cycle metabolites for sepsis diagnosis (Table 1), septic shock diagnosis (Table 2), prognostication of sepsis (Table 3), prognostication of septic shock (Table 4), and monitoring the treatment response (Table 5). A down-sided triangle (▼) represents a decreased level, whereas an up-sided triangle (▲) represents the opposite. The alterations of several sugars are found, including glucose, sucrose, mannose, and myo-inositol. Glucose, a main energy source for human cells, converts to pyruvate and acetyl-CoA, respectively, before entering the TCA cycle. In addition, acetylcarnitine (C2 carnitine) can feed via acetyl-CoA as well. Another anaerobic metabolite, lactate, are found to be increased in sepsis patients with poor prognosis. Citrate is the initial metabolite of the TCA cycle, which turns into isocitrate, α-ketoglutarate, succinyl-CoA, succinate, fumarate, malate, and oxaloacetate, respectively. The increases in TCA cycle-related metabolites represent an augmentation of aerobic metabolism during sepsis, which can promote oxidative phosphorylation and reactive oxygen species (ROS) production. An increase in the ROS level may be one of the potential mechanisms mediating sepsis-induced mitochondrial dysfunction.

Besides hemodynamic resuscitation in sepsis patients, mitochondrial resuscitation remains another issue for treatment response monitoring [101]. Although lactate is a commonly accepted use for the monitoring of tissue perfusion, the alternations of mitochondrial TCA cycle metabolites may also be used in mitochondrial resuscitation as well. A complete understanding of whole glycolysis and TCA cycle metabolites, as in Figure 4, could be well-achieved by metabolomics study.

## 9. Limitation and Future Direction of the Metabolomic Research in Sepsis

The current advanced technology of metabolomics is just shinning the light at the beginning of the tunnel in terms of providing more precise biomarkers for sepsis. An uncertain standardization of the metabolomic studies is one of the major issues for replicating the results. Moreover, the complexity and heterogeneity of sepsis create are a large variety of study populations. Therefore, a combination of conventional biomarkers and metabolomic profiling is likely a key solution. Moreover, multi-omics modalities and systems of biology should be simultaneously approached, since sepsis is also associated with the alterations of protein and gene expression [102,103].

According to septic shock resuscitation, besides the bed-sided hemodynamic monitoring, metabolic and cellular resuscitation are crucial strategic keys to improve patient outcomes. Currently, no definite metabolic abnormality is warranted beyond hyperlactatemia [1]. We highly expect that metabolomics can be one of the solutions used to examine mitochondrial function or even abnormalities of the microcirculation in sepsis. Future studies with the metabolomics approach, attempting to develop bedside laboratory kits, are incredibly significant for the clinical practice.

Metabolomics to foresee outcomes of patients with sepsis and septic shock are undergoing research. Death signaling metabolites’ modulation is another remedy to improve patients’ outcomes. The integral application of pharmacometabolomics to identify the appropriate drugs or to certify suitable, well-responsive patients is another approach for precision medicine.

Nowadays, a lot of information about metabolites in sepsis comes across without enough pathophysiological elucidation. Moreover, the overwhelming evidence of metabolomic information in sepsis is challenging. Therefore, artificial intelligence facilitating machine learning experiences may be a worthwhile solution to handle this enormous information. In the end, we believe that metabolomics can illuminate the light at the end of the tunnel of sepsis management.

## Figures and Tables

**Table 1:** Metabolomics-assisted diagnosis of sepsis in critically ill patients.

Age (Sample Size)	APACHE-II Score	Samples Since Admission			Methods	Major Findings in Sepsis Group			Interpretation	Citation
		Serum	Plasma	Others		Metabolic Pathways	Decreased	Increased		
N/A (102) vs. N/A (56)	N/A vs. N/A		✓ D1		Targeted (LC-MS/MS)	At D1			Patients with sepsis had increased ceramides, but decreased phospholipids when compared to patients without sepsis	[8]
						Fatty acids	Ceramides C23:0, C24:0	Ceramides C16:0, C18:0, C20:0, C22:1, C24:1, and total form		
						Phospholipids	LysoPC			
T: 64 ± 11 (30) vs. 67 ± 10 (33) V: 64 ± 15 (39) vs. 67 ± 10 (41)	23 ± 8 vs. 18 ± 7 26 ± 9 vs. 19 ± 7		✓ within 24 h/ at onset of SIRS		Targeted (LC-MS/MS)	Fatty acids	-	AC C3, C5, C6 (C4:1-DC), C8, C10:1	Fatty acids and phospholipids are potential markers for discriminating sepsis from SIRS	[9]
						Phospholipids	-	PCaa PCae		
64 ± 17 (35) vs. 59 ± 19 (15)	22 ± 7 vs. 11 ± 9	✓ within 24 h			Targeted (LC-MS/MS)	Amino acids and amine	*S*-phenyl-d-cysteine	*N*-nonanoyl glycine	Amino acids and lactitol dihydrate could differentiate sepsis from SIRS	[10]
						Others	Lactitol dihydrate	*S*-(3-methyl-butanoyl)-dihydrolipoamide-E		
57 ± 22 (35) vs. 47 ± 13 (14)	18 ± 8 vs. 9 ± 3	✓ within 24 h			Targeted (LC-MS/MS)	Amino acids and amine	Anserine Lysine Phosphoethano-lamine δ-Hydroxylysine	Ethanolamine Homocitrulline Cystathionine	Critically ill patients with sepsis had a wide range of amino acid spectral changes that differ from SIRS	[11]
T: 70 ± 17 (123) vs. 63 ± 16 (42) V: 64 ± 24 (59) vs. 60 ± 18 (2)	11 ± 6 vs. 11 ± 8 13 ± 11 vs. 9 ± 6	✓ within 24 h			Targeted (LC-MS/MS)	Amino acids and amine	-	Serine Aspartate Phenylalanine Dimethylarginine Acetylornithine Kynurenine Spermine Spermidine	Amino acids, fatty acids, and phospholipids can potentially be used as sepsis biomarkers	[12]
						Fatty acids	AC C16:2(OH)	AC C6(C4:1-DC)		
						Phospholipids	PCaa C32:2, C36:6, C40:4, C42:6 PCae LysoPCa SM C20:2, C22:3, C24:0, C26:1 SM-OH C22:1, C24:1	PCaaC32:0 SM C16:1		
56 ± 18 (20) vs. 58 ± 11 (20)	15 ± 6 vs. N/A		✓ within 36 h		Targeted (LC-MS and GC-MS)	Phospholipids	LysoPC PC C16:0/20:1, C16:0/20:3 SM	PC C15:0/18:2, C16:0/18:1, C16:0/18:2, C16:0/20:5	Fatty acids and phospholipids detected in plasma and erythrocytes could signal sepsis vs. non-sepsis	[13]
				Erythrocytes ✓ within 36 h	GC-MS	Fatty acids	n-3 PUFA DPA (C22:5 n-3) DHA (C22:6 n-3)	Total MUFA Oleic (C18:1 n-9)		
						Phospholipids	LysoPC PC C15:0/18:2 SM	PC C16:0/18:2, C16:0/20:4, C16:0/20:5 PS		

Continuous data are presented in mean ± SD; N/A not available. Abbreviations: a, acyl; aa, diacyl; ae, acyl-akyl; AC, acylcarnitine; APACHE-II score, Acute Physiology and Chronic Health Evaluation-II score; C, number of carbons in the fatty acid side chain; DHA, docosahexaenoic acid; DPA, docosapentaenoic acid; LysoPC, lysophosphatidylcholine; LC-MS/MS, liquid chromatography-tandem mass spectrometry; MUFA, monounsaturated fatty acid; PC, phosphatidylcholine; PS, phosphatidylserine; PUFA, polyunsaturated fatty acid; SM, sphingomyelin; SIRS, systemic inflammatory response syndrome; T, training dataset; V, validation dataset.

**Table 2:** Metabolomics-assisted diagnosis of septic shock in critically ill patients.

Age (Age Range) (Sample Size)	APACH-II Score	Samples Since Admission		Methods	Major Findings in Septic Shock Groups			Interpretation	Citation
		Serum	Plasma		Metabolic Pathways	Decreased	Increased		
62 (55–73) (39) vs. 66 (56–71) (20)	23 (16–31) vs. 14 (13–17)	✓ within 24 h		Targeted (^1^H-NMRS)	Amino acids and amines	Isoleucine Glutamine Alanine Leucine Lysine Glycine Serine Threonine Valine Glutamate Arginine 2-Aminobutyrate	Phenylalanine 2-Hydroxy-isovalerate Proline Trimethylamine *N*-oxide	Septic shock patients had different patterns in amino acids, fatty acids, and TCA cycle metabolites	[23]
					Fatty acids	-	Isobutyrate		
					Glycolysis	Glucose Mannose	Lactate Sucrose Myoinositol AC C2		
					TCA cycle	-	Succinate		
62 (56–73) (37) vs. 66 (56–71) (20)	23 (16–31) vs. 14 (13–17)	✓ within 24 h	✓ within 24 h	Targeted (^1^H- NMRS)	Amino acids and amines	Threonine Valine Arginine Glutamate	Phenylalanine Proline	Septic shock patients had different patterns of metabolites, particularly amino acids	[42]
					Glycolysis	Glucose	Myoinositol AC C2		

Continuous data are presented in median (IQR1-3). Abbreviations: ^1^H-NMRS, ^1^H-Nuclear Magnetic Resonance Spectroscopy; APACHE-II score, Acute Physiology and Chronic Health Evaluation-II score; TCA, tricarboxylic Acid.

**Table 3:** Metabolomics-assisted prognostication of patients with sepsis non-survivors.

Settings	Age (Age Range) (Sample Size)	APACHE-II Score	Samples Since Admission			Methods	Major Findings in Non-Survivors			Interpretation	Citation
			Serum	Plasma	Blood		Metabolic Pathways	Decreased	Increased		
48-H mortality	67 ± 15 (9) vs. 63 ± 18 (26)	26 ± 6 vs. 20 ± 8	✓ within 48-H before death			Targeted (LC-MS/MS)	Amino acids and amines	-	*S*-(3-methyl-butanoyl)dihydro-lipoamide-E	Amino acids and phospholipids could indicate the possibility of death within 48-H in patients with sepsis	[10]
							Phospholipids	-	Phosphatidyl-glycerol(22:2 (13Z,16Z)/0:0) GPC		
7-D mortality	60 (36–80) (9) vs. 60 (27–84) (13)	31 (16–46) vs. 22 (14–38)		✓ within 48 h = D1, D3, and D7		Targeted (LC-MS/MS)	Persisted D1 to D7			Fatty acids and proresolving lipids signal 7-D mortality in critically-ill patients with sepsis	[60]
							Fatty acids	-	Prostaglandin F2α Leukotriene B4 Resolvin E1 Resolvin D5 17R-Protectin D1		
							At D1				
							Fatty acids	-	7-HDHA 17-HDHA 15-HEPE 18-HEPE 15-HETE		
								-	Protectin D1		
							At D3				
							Fatty acids	-	17-HDHA 18-HEPE 5-HETE 15-HETE 5S,12S-diHETE		
								-	17-epi-Resolvin D1 17-epi-Protectin D1		
							At D7				
							Fatty acids	-	4S,14S-diHDHA 5S,15S-diHETE		
								-	Resolvin E2		
28-D mortality	69 ± 17 (31) vs. 56 ± 19 (90)	23 ± 8 vs. 15 ± 7		✓ H0 and H24	✓ H0 and H24	- Untargeted (UPLC-MS/MS and GC-MS) - Targeted (UPLC-MS/MS)	Persisted At H0 to H24			28-D mortality could be predicted by several amino acids, amines, fatty acids, and glycolysis metabolites	[61]
							Amino acids and amines	-	*N*-Acetylthreonine 1-Methylimidazole acetate *N*-Acetylalanine Hydroxyproline Prolylhydroxy-proline SDMA AC C3, C4, C5, C5:1		
							Fatty acids	-	AC C6, C8, C10, C16, C18 3-Hydroxy-2-ethyl-propionate		
							Glycolysis	-	Erythronate *N*-acetylneura-minate Arbitol AC C2		
							TCA cycle	-	Malate		
							At H0				
							Fatty acids	-	AC C12		
							At H24				
							Amino acids and amines	-	α-Hydroxyiso-valerate Glutaroylcarnitine Hydroxyisovalero-ylcarnitine		
							Fatty acids	-	Hexadecanedioate		
							Phospholipids	1-Arachido-nyl-GPE 1-Palmitoyl-GPC 1-Stearoyl-GPC 1-Eicosatri-enoyl-GPC 1-Arachido-noyl-GPC	-		
28-D mortality	T: 58 ± 15 (30) vs. 53 ± 14 (60) V: 69 ± 16 (34) vs. 58 ± 17 (115)	30 ± 11 vs. 23 ± 9 23 ± 8 vs. 15 ± 7		✓ H0		Targeted (GC-MS and LC-MS)	Amino acids and amines	-	Ornithine Kynurenine AC C3, C4, C5, C5-OH, C5:1 β-hydroxyiso-valerate *N*-acetylalanine *N*-acetylserine α-Hydroxy-isovalerate γ-glutamylphenyl-alanine γ-glutamyltyrosine	Non-surviving 28-D sepsis patients had specific changes in amino acids, fatty acids, glycolysis, and bile acids’ metabolic pathways, as well as an increase in aromatic microbial metabolites	[62]
							Fatty acids	-	AC C6		
							Phospholipids	1-arachidonyl-GPE (20:4) 1-arachidonyl-GPC (20:4) 1-linoleoyl-GPC (18:2) 2-palmitoyl-GPC (16:0) 1-palmitoyl-GPC (16:0) 1-stearoyl-GPC (18:0)	-		
							Glycolysis Aromatic microbial metabolites	- -	Sucrose Lactate 3-(4-hydroxyphenyl) lactic acid		
28-D mortality	61 ± 21 (15) vs. 54 ± 23 (20)	22 ± 8 vs. 10 ± 5		✓ within 24H = D1, D3, D5, D7, D10, and D14		Targeted (LC-MS/MS)	At certain time points			Amino acids could indicate the possibility of death in septic patients	[11]
							Amino acids and amines	Arginine Glutamate Serine Phosphoserine Taurine Tryptophan	α-Aminoadipic acid Cystathionine Ethanolamine Phenylalanine		
28-D mortality	68 (51–75) (31) vs. 63 (53–74) (89)	12 (8–9) vs. 9 (6–13)		✓ within 24H = D1, D3, D7		Targeted (LC-MS/MS)	Amino acids and amines		SDMA ADMA	High level of plasma SDMA and ADMA can predict sepsis non-survival	[66]
28-D mortality	70 ± 13 (21) vs. 72 ± 15 (69)	26 ± 9 vs. 23 ± 8		✓ H0		Targeted (UHPLC-MS)	Glycolysis	-	AC C2	Acetylcarnitine can forecast 28-D mortality in patients with sepsis	[63]
28-D mortality	67 ± 14 (54) vs. 62 ± 19 (134)	22 (18–30) vs. 18 (13–24)		✓ H0		Targeted (LC-MS)	Amino acids and amines	-	Isoleucine Alanine	Particular metabolites can forecast 28-D mortality in sepsis patients	[64]
							Phospholipids	LysoPC C22:0 LysoPC C24:0	-		
							Glycolysis	-	Lactate Pyruvate AC C2		
30-D mortality	55 (17–80) (39) vs. 54 (20–91) (63)	N/A vs. N/A		✓ D1, D4, and D11		Targeted (LC-MS/MS) Lipids	Persisted along D1 to D11				[8]
							Fatty acids and phospholipids	-	Total ceramides-to-SM ratio/total LysoPC-to-PC ratio		
90-D mortality	75 ± 13 (30) vs. 71 ± 13 (63)	9 ± 4 ^$^ vs. 5 ± 4 ^$^			✓ D1	Targeted (UPLC-MS)	Amino acids and amine	-	Phenylalanine Leucine	In sepsis patients, 90-D mortality can be expected by phenylalanine and leucine	[65]

Continuous data are presented in mean ± SD, otherwise reported as median and IQR1-3. ^$^ Sequential Organ Failure Assessment (SOFA) score. Abbreviations: 7-HOCA, 7-α-hydroxy-3-oxo-4-cholestenoate; APACHE-II score, Acute Physiology and Chronic Health Evaluation-II score; ADMA, asymmetric dimethylarginine; C, number of carbons in the fatty acid side chain; D, Day; GPC, Glycerophosphocholine; GPE, Glycerophosphoethanolamine; H, Hour; HDHA, Hydroxydocosahexaenoate; HEPE; Hydroxyeicosapentaenoate; HETE; Hydroxyeicosatetraenoate; LysoPC, lysophosphatidylcholine; LC-MS/MS, liquid chromatography-tandem mass spectrometry; SDMA, symmetric dimethylarginine; UHPLC-MS, Ultra-high performance liquid chromatography mass spectrometry; UPLC-MS, Ultra-performance liquid chromatography-mass spectrometry; T, training cohort; V, validation cohort.

**Table 4:** Metabolomics-assisted prognostication of patients with septic shock non-survivors.

Settings	Age (Age Range) (Sample Size)	APACHE-II Score	Samples Since Admission			Methods	Major Findings in Non-Survivors (NS)			Interpretation	Citation
			Serum	Plasma	Urine		Metabolic Pathways	Decreased	Increased		
ICU mortality	63 (60–77) * (8) *	26 (18–31) *	✓ within 24 h	✓ within 24 h		Targeted (^1^H-NMRS)	Amino acids and amines	Dimethylamine	-	Non-survivors in septic shock had high levels of 2-Hydrocyiso-valerate and fructose	[42]
							Fatty acids	-	2-Hydroxyiso-valerate		
							Glycolysis	-	Fructose		
24-H mortality	72 ± 0.4 (30) vs. 69 ± 0.3 (40)	12 ± 0.6 ^$^ vs. 11 ± 0.7 ^$^	✓ H0 and H24 of vaso-pressor initiation			Targeted (^1^H-NMRS)	At H0;			Non-surviving patients with 24-H septic shock can be forecasted by amino acids, TCA cycle metabolites, and fatty acids pathways	[86]
							Amino acids and amines	-	Alanine Glutamine Glutamate Methionine Phenylalanine Tyrosine Lysine 1-Methylhistidine		
							Glycolysis	-	Pyruvate Lactate		
							TCA cycle	-	Citrate Fumarate		
							At H24;				
							Amino acids and amines	-	Tyrosine Phenylalanine Glutamine Glutamate Alanine 1-Methylhistidine		
							Fatty acids	-	2-Hydroxyiso-valerate		
							Glycolysis	-	Lactate		
							TCA cycle	-	Citrate Pyruvate		
							∆H24-H0 in nonsurvivors;				
							Amino acids and amines	-	Glutamate Glutamine Phenylalanine Alanine		
							Glycolysis	-	Pyruvate Lactate		
							TCA cycle	-	Citrate		
							Others	*N*-Acetyl-glycoprotein	Creatinine		
							∆H24-H0 in survivors;				
							Amino acids and amines	Alanine Glutamine Phenylalanine	-		
							Glycolysis	Lactate	-		
							TCA cycle	Pyruvate Citrate	-		
7-D mortality	66 ± 1 (21) vs. 64 ± 1 (29)	68 ± 2 ^#^ vs. 54 ± 2 ^#^	✓ H0			Untargeted (UPLC-MS)	Amino acids and amines	Ornithine Arginosuccinate Citrulline	Proline Valine Leucine Isoleucine Glutamine Glutamate Phenylalanine Betaine	Non-surviving 7-D septic shock patients demonstrated several precise metabolomics signals from amino acids, TCA cycle, fatty acids, and phospholipids pathways	[87]
							Fatty acids	AC C16, C18	AC C6, C10, C12		
							Phospholipids	LysoPE	LysoPC		
							Glycolysis	AC C2	Lactate		
							TCA cycle	-	Succinate Malate α-ketoglutarate Citrate		
28-D mortality	70 ± 12 (11) vs. 61 ± 15 (9)	12 ± 2 vs. 11 ± 2		✓ D1 and D7		Targeted (LC-MS/MS)	At D1			Long chain PC and LysoPC metabolites had predictive capability for 28-D mortality patients in septic shock	[88]
							Phospholipids	LysoPCa PCaa C38:6 PCae SM	PCaa C30:2, C38:1		
							Glycolysis	AC C2	-		
							At D7				
							Amino acids and amines	-	Kynurenine		
							Phospholipids	LyscoPCa PCaa PCae	-		
							∆D7-D1 comparing between NS vs. S				
								↔ vs. ↓			
							Amino acids and amines	Kynurenine	-		
								↔ vs. ↑	↑ vs. ↑↑		
							Phospholipids	LysoPCa PCae C34:3	PCae C32:2		
								↓vs. ↔			
							Phospholipids	PCaa	-		
28-D mortality	64 ± 17 (8) vs. 66 ± 14 (9)	D1: 12 ± 3 ^$^ vs. 11 ± 2 ^$^ D7: 9 ± 5 ^$^ vs. 5 ± 2^$^		✓ at Shock Dx		Targeted (LC-MS/MS)	Crude ratio of D7/D1			The ratios of particular amino acids and phospholipids can determine 28-D mortality in septic shock patients	[89]
							Amino acids and amines	-	SDMA Total DMA		
							Phospholipids	LysoPCa C24:0	PCaa		
							Ratio of D7/D1 discriminated by multivariate analysis				
							Amino acids and amines	Methionine	Proline Tyrosine		
							Phospholipids	PCaa C40:6, C42:6, C42:2 PCae C30:2, C42:5 LysoPCa	PCaa C34:3, C36:3, C36:6, C42:1, C42:5 PCae C30:1		
30-D mortality	65 (37–79) (12) vs. 60 (24–80) (48)	21 ± 5 vs. 19 ± 6			✓ H0 and H24	Untargeted (^1^H-NMRS)	Amino acids and amines	Methionine Glutamine Arginine Phenylalanine	-	Particular amino acids, glycolytic metabolites, and alcohol can predict 30-D mortality in septic shock patients	[90]
							Glycolysis	-	Glucose		
90-D mortality	70 ± 12 (11) vs. 61 ± 15 (9)	12 ± 2 vs. 11 ± 2		✓ D1 and D7		Targeted (LC-MS/MS)	At D1			Long chain PC and LysoPC metabolites had predictive capability for 90-D mortality in septic shock patients	[88]
							Phospholipids	PCaa C36:6, C38:4, C38:6	-		
							At D7				
							Phospholipids	LysoPCa C16:0, C16:1, C18:0 PCaa PCae	LysoPCa C24:0		
							∆D7-D1 comparing between NS vs. S				
								↔ vs. ↓			
							Amino acids and amines	Kynurenine	-		
								↔ vs. ↑	↑ vs. ↑↑		
							Phospholipids	LysoPCa PCaa C32:3	PCaa C34:3, C34:4 PCae C32:2		
								↔ (↓) vs. ↔ (↑)			
							Phospholipids	PCaa C38:1	-		
1-Y mortality	69 (61–77) (4) vs. 58 (50–65) (7)	15 (14–17) ^$^ vs. 14 (9–14) ^$^	✓ H0, H24, and H48 after l-carnitine infusion			Untargeted (LC-MS)	Amino acids and amines	*N*-acetyl-l-phenylalanine Phenylalanyl-tyrosine	*N*-methyl-phenyl-alanine Isoleucyl-proline Leucyl-proline	1-Y mortality in septic shock patients can be determined by certain amino acids, fatty acids, and peptide/short chain proteins	[91]
							Fatty acids	-	Adipoyl-l-carnitine		

Continuous data are presented in mean ± SD, otherwise reported as median and IQR1-3. ^$^ Sequential Organ Failure Assessment (SOFA) score. ^#^ Simplified Acute Physiology score 2 SAPS2) * overall information. Abbreviations: ^1^H-NMRS, ^1^H-Nuclear Magnetic Resonance Spectroscopy; a, acyl; aa, diacyl; ae, acyl-akyl; APACHE-II score, Acute Physiology and Chronic Health Evaluation-II score; C, number of carbons in the fatty acid side chain; D, Day; DMA, Dimethylarginine; GCA, Glycocholic acid; GCDCA, Glycochenodeoxycholic acid; GUDCA, Glycoursodeoxycholic acid; GUDCS, 3-glycine chenodeoxycholic acid; H, Hour; LC-MS, liquid chromatography-mass spectrometry; LC-MS/MS, liquid chromatography-tandem mass spectrometry; LysoPC, lysophosphatidylcholine; LysoPE, Lysophosphatidylethanolamine; NS, Non-survivor; S, Survivor; SDMA, Symmetric dimethylarginine; SM, Sphingomyelin; TCA cycle, tricarboxylic acid cycle; UDCA, Ursodeoxycholic acid; UPLC-MS, Ultra-performance liquid chromatography-mass spectrometry; Y, Year.

**Table 5:** Metabolomics-assisted treatment monitoring in patients with sepsis/septic shock.

Settings	Studies Groups	Age (Age Range) (Sample Size)	APACHE- II Score	Samples		Methods	Major Findings in Responder Groups			Interpretation	Citation
				Serum	Plasma		Metabolic Pathway	Decreased	Increased		
l-carnitine responders vs. placebo in septic shock patients treated with vasopressor	Low ketones vs. High ketones categorized by 3-hydroxy-butyrate (cut-off= 153 μM)	60 (52–68) (15) vs. 69 (60–74) (15)	10 (9–14) ^$^ vs. 13 (8–14) ^$^	✓ H0, H24, and H48 after l-carnitine infusion		Untargeted (^1^H-NMRS)	At H24			Pharmacometa-bolomics can be used to guide responses to l-carnitine treatment	[99]
							Amino acids and amines	-	Methionine Lysine Phenylalanine Tyrosine		
							Fatty acids	Carnitines	-		
							Glycolysis	AC C2	-		
							At H48				
							Fatty acids	Carnitines	-		
							Glycolysis	AC C2	-		
Characterized response to therapy in patients with septic shock	Response (R) vs. Non-response (NR) to therapy	67 (61–75) (14) vs. 75 (66–82) (7)	35 (31–38) vs. 38 (37–39)		✓ H0 and H48 after resus-citation	Untargeted (LC-MS/MS)	At H0, R vs. NR			Metabolomics from particular pathways including amino acids, fatty acids, phospholipids, and TCA cycle had potential roles for treatment monitoring in patients with septic shock	[100]
							Amino acids and amines	Histidine	-		
							Fatty acids	-	Stearic acid		
							Glycolysis	Pyruvate Lactate	-		
							In R, H48 vs. H0;				
							Amino acids and amines	Taurine	Threonine Tyrosine Lysine Kynurenine		
							Fatty acids	Acetylcarnitine Myristic acid Palmitoleic acid Palmitic acid Oleic acid	-		
							TCA cycle	Citrate	-		
							In NR, H48 vs. H0;				
							Amino acids and amines	-	Threonine Arginine Lysine		
							Fatty acids	Acetylcarnitine Stearic acid Myristic acid Palmitoleic acid Palmitic acid Oleic acid	-		
							Comparing R vs. NR overtime				
								↓ vs. ↓↓	↓ vs. ↑		
							Fatty acids	Myristic acid Oleic acid			
						Targeted (LC-MS)	In R, H48 vs. H0;				
							Amino acids and amines	Histidine Taurine	Arginine Lysine Ornithine Serine Threonine Tryptophan Tyrosine Methionine sulfoxide		
							Phospholipids	-	SM LysoPCa PCaa PCae		
							In NR, H48 vs. H0;				
							Amino acids and amines	Taurine	Arginine Lysine Ornithine Serine Threonine Tryptophan Kynurenine		
							Phospholipids	SM(OH) C24:1 PCaa PCae	LysoPCa		
							Comparing R vs. NR overtime;				
								↑ vs. ↑↑			
							Amino acid and amines	Kynurenine	-		
								↑ vs. ↓	↑↑ vs. ↑		
							Phospholipids	SM C16:0, C18:0, C18:1 SM(OH) C24:1 PCaa PCae	SM C16:1 LysoPCa		
							At H48, R vs. NR;				
							Amino acids and amines	Alanine Histidine Methionine Phenylalanine	Glutamate		
							Phospholipids	-	SM LysoPCa PCae		

Continuous data are presented as median and IQR1-3. ^$^ Sequential Organ Failure Assessment (SOFA) score; a, acyl; aa, diacyl; ae, acyl-akyl; APACHE-II score, Acute Physiology and Chronic Health Evaluation-II score; C, number of carbons in the fatty acid side chain; H, Hour, LC-MS, liquid chromatography-mass spectrometry; LC-MS/MS, liquid chromatography-tandem mass spectrometry; LysoPC, lysophosphatidylcholine; NR, Non-responder; PC, phosphatidylcholine; R, Responder; SM, Sphingomyelin; TCA cycle, tricarboxylic acid cycle.
